# Caught in Action: Selecting Peptide Aptamers Against Intrinsically Disordered Proteins in Live Cells

**DOI:** 10.1038/srep09402

**Published:** 2015-03-24

**Authors:** Jacqueline D. Cobbert, Christopher DeMott, Subhabrata Majumder, Eric A. Smith, Sergey Reverdatto, David S. Burz, Kathleen A. McDonough, Alexander Shekhtman

**Affiliations:** 1 Department of Chemistry, University at Albany, Albany, NY; 2 Wadsworth Center, NY State Department of Health, Albany, NY

## Abstract

Intrinsically disordered proteins (IDPs) or unstructured segments within proteins play an important role in cellular physiology and pathology. Low cellular concentration, multiple binding partners, frequent post-translational modifications and the presence of multiple conformations make it difficult to characterize IDP interactions in intact cells. We used peptide aptamers selected by using the yeast-two-hybrid scheme and in-cell NMR to identify high affinity binders to transiently structured IDP and unstructured segments at atomic resolution. Since both the selection and characterization of peptide aptamers take place inside the cell, only physiologically relevant conformations of IDPs are targeted. The method is validated by using peptide aptamers selected against the prokaryotic ubiquitin-like protein, Pup, of the mycobacterium proteasome. The selected aptamers bind to distinct sites on Pup and have vastly different effects on rescuing mycobacterial proteasome substrate and on the survival of the Bacille-Calmette-Guèrin, BCG, strain of *M. bovis*. This technology can be applied to study the elusive action of IDPs under near physiological conditions.

On average 4% of prokaryotic and 33% of eukaryotic genomes encode proteins or large segments of proteins that lack a stable secondary or tertiary structure^1^. These intrinsically disordered proteins (IDPs) or disordered regions can be highly conserved between species, and are often functional^2^,^3^,^4^. IDPs have the ability to bind to multiple proteins and play a role in the assembly of macromolecular arrays^5^,^6^. IDPs are implicated in neuropathology, autoimmune diseases and cancers, and are considered to be promising targets for drug therapy^7^. Despite wide recognition of IDPs and the nascent secondary structures that exist in the unbound state, the molecular mechanisms underlying the functions of IDPs are not widely understood.

There is a limited arsenal of molecular tools that can be applied to study interactions of IDPs in a physiological milieu^5^,^8^,^9^. Due to the intrinsic flexibility of IDPs, the powerful methodology of using small molecules to block protein-protein interaction surfaces are applied to well-folded IDP binding partners; there is only one example of small molecule inhibitor to IDP, c-Myc^10^. Since many IDPs fold upon binding to their cognate targets^4^,^5^,^11^, site-directed mutagenesis, which creates local changes in the chemical structure of the IDP, cannot be effectively used to interrogate the usually large IDP-target interaction surfaces^5^,^12^.

Recently we introduced a Combinatorial Library of Improved Peptide aptamers (CLIPs), containing more than 3*10^10^ peptide aptamers, as a tool to help isolate site-specific binders to select cellular targets^13^. Peptide aptamers^14^,^15^ (PA) are small ~12 kDa proteins that consist of a randomized 8 amino acid peptide sequence inserted into the loop of a modified thioredoxin platform. The modification of the thioredoxin platform^13^ improves solubility and decreases the tendency of PAs to oligomerize, creating molecules with drug-like binding abilities^16^.

PAs are selected by using the yeast-two-hybrid (Y2H) technique^14^,^15^, which facilitates binding to a broad range of cellular, viral and bacterial target proteins with high specificity and strong affinity^16^. CLIPs technology is an attractive molecular tool to analyze the functional consequences of nascent structures present in free IDPs due to the *in vivo* nature of target selection and comprehensive coverage of the potential binding surfaces.

In-cell NMR is used to analyze nascent structures of IDPs inside live cells at atomic resolution^8^,^17^,^18^,^19^,^20^,^21^. STINT-NMR is an in-cell technique developed to study protein-protein structural interactions inside live cells^22^,^23^. We used STINT-NMR to define the interaction surfaces between a small IDP, the prokaryotic ubiquitin-like protein, Pup^24^, and selected PAs, in the crowded cytosol. Unlike its eukaryotic counterpart, Pup is unstructured^25^. Since both selection and structural characterization of PA-IDP complexes are made inside cells, we expect that this technology will be highly relevant to identify functional complexes between IDPs and its binding targets.

The *Mycobacterium tuberculosis* (Mtb) proteasomal system is an attractive target for drug therapy against latent Mtb infection since it provides defense against the reactive nitrogen intermediates generated to counter bacteria inside phagosomes^26^,^27^. Indeed, the potent proteasome inhibitors, Bortezomib^28^ (Btz) and GL5^27^, increase the sensitivity of Mtb to nitro-oxidative stress^27^. The Mtb proteasome system consists of the Mtb core particle, CP, a proteasomal ATPase, Mpa, and prokaryotic ubiquitin-like protein, Pup. Mpa binds to the proteasome CP and Pup binding to Mpa directs the pupylated substrate into the proteasome for degradation. We used the Mtb proteasome system to illustrate how the combination of CLIPs and STINT-NMR technologies allows the selection of specific binders to Pup and how these molecules can be used to analyze the functional interactions of Pup inside mycobacteria.

## Results

### Selection of peptide aptamers that interact with Pup

**Figure 1:**
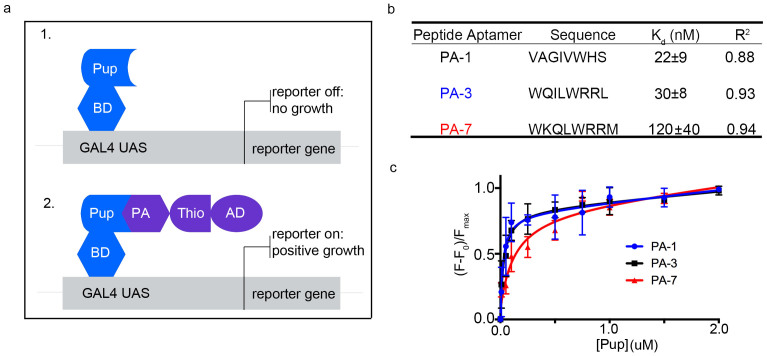
Y2H selected PAs bind to Pup with nanomolar affinity. (a) Yeast-Two-Hybrid selection of PAs. 1) Pup, fused to the Gal4 DNA-binding domain (BD), is expressed in yeast containing several reporter genes that possess sites for the BD in their promoters. The reporter genes are not expressed if the activation domain (AD) is not present. 2) Pup, fused to the BD, is expressed concurrently with a randomized PA loop in a thioredoxin (Thio) scaffold^13^, fused to the AD. Binding between Pup and a PA activates transcription of reporter genes in cells, while reporter gene transcription is suppressed in cells where Pup and a PA do not interact. (b) PA sequences selected through Y2H screening and the Pup-binding affinity of each sequence. (c) Pup-PA binding isotherms. Changes in native PA fluorescence intensity are plotted vs. the concentration of Pup. Thio scaffold^13^ was used as a negative control and showed no changes in fluorescence upon titration with Pup. The data represent mean values and are fitted as described in Methods. All experiments were performed in triplicate.

Yeast two-hybrid selection (Fig. 1a) was used to identify PAs that bind specifically to Pup *in vivo*. Pup fused to the Gal4 DNA binding domain (BD) was used as bait, and a randomized PA library, CLIPs^13^, was the prey. *E. coli* thioredoxin, fused to the GAL4 transcriptional activation domain (AD), served as the scaffold to constrain the octa-peptides of randomized sequence in an aptamer loop^13^,^14^. Approximately 1.5*10^7^ transformants were screened on Ade^−^ plates and out of fifty selected, three clones, designated PA-1, PA-3, and PA-7, were found to activate all Y2H markers, *ADE2, HIS3, MEL1*, and *AUR1-*C, indicating molecular interactions between PAs and Pup.

DNA encoding the interacting PAs was PCR amplified from the yeast and sequenced (Fig. 1b). PA-1 contains primarily nonpolar aliphatic and two aromatic residues, tryptophan and histidine. PA-3 and PA-7 are similar in sequence, and contain positively charged and aromatic residues. To estimate the binding affinity of each PA for Pup *in vitro*, fluorescence titrations were conducted using bacterially over-expressed and purified components. Fluorescence signals were quenched as Pup was added (Fig. 1c). In contrast, the thioredoxin scaffold^13^, which was used as a negative control, did not show fluorescence quenching upon adding Pup. The resolved K_d_'s are in the nanomolar range.

### Peptide aptamers bind to the α-helix and N-terminal region of Pup

**Figure 2:**
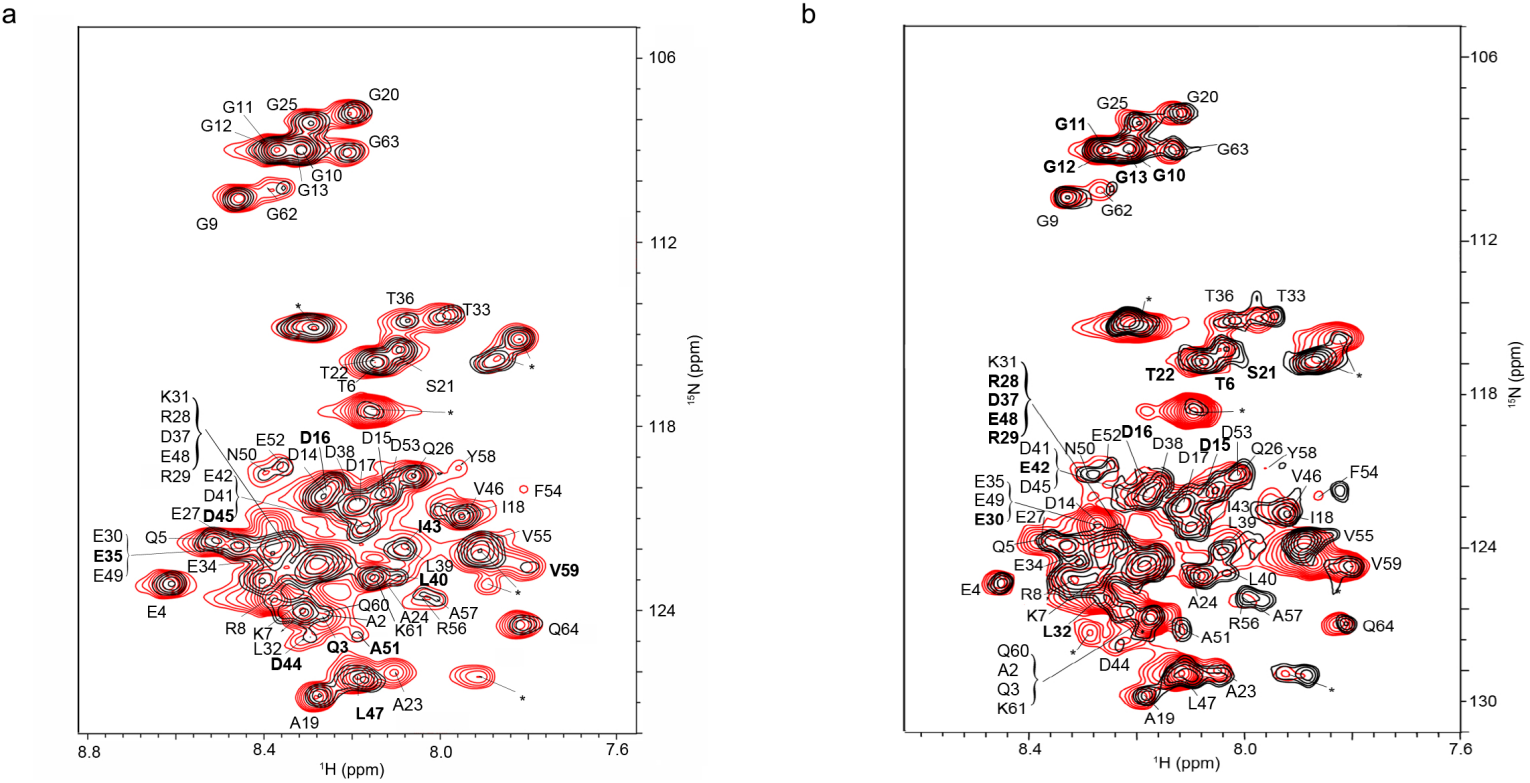
In-cell interaction of Pup and peptide aptamers, PA-1 and PA-3. ^1^H{^15^N}-HSQC spectra of *E. coli* after 2 h of [*U*-^15^N] Pup over-expression (red), overlaid with spectra (black) of *E. coli* after 2 h of [*U*-^15^N] Pup over-expression followed by approximately 16 h over-expression of PA-1 (a) or PA-3 (b). Due to ^15^N editing, only backbone amide protons and nitrogens of Pup are present in the spectra. Most peaks do not change their positions indicating that only a subset of Pup residues interact with each PA. Pup residues identified by SVD analysis as involved in the interaction with PAs are in bold. The sharp peaks in the spectra, which correspond to various metabolites of [*U*-^15^N] ammonium ion, are labeled with asterisks. Changes in metabolite peak intensities indicate overall changes in cell metabolism over the course of protein overexpression.

We used in-cell NMR (STINT-NMR) to identify the Pup residues affected by PA binding^22^. [*U*-^15^N]-Pup was overexpressed in *E. coli* BL21(DE3) for two hours, followed by overexpression of unlabeled PA for up to 16 hours; spectra were collected at time intervals corresponding to increasing PA concentrations. After collecting the sample, the cells were subjected to in-cell NMR analysis without prior freezing. The in-cell ^1^H{^15^N}-HSQC NMR spectrum of free Pup was used as a control (Fig. 2 and Supplementary Fig. 1). The acquisition time for an in-cell NMR experiment was less than two hours. To verify that the protein signals originated from cellular protein, after each in-cell NMR experiment, the cells were recovered from the tube, centrifuged, and the NMR spectrum of the supernatant was recorded; no protein signals were detected.

**Figure 3:**
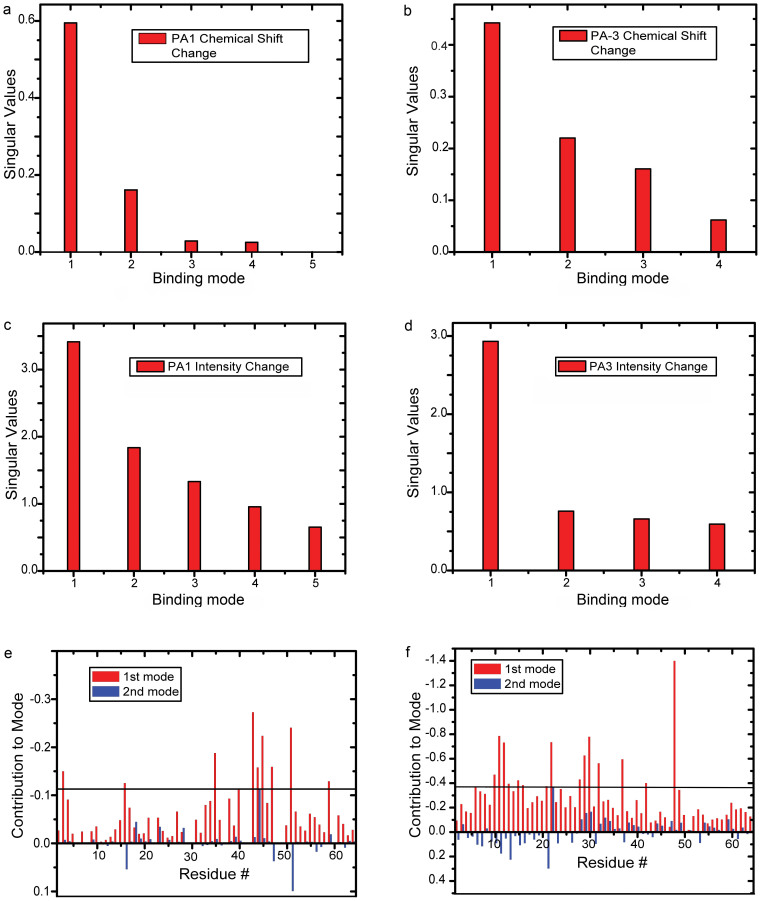
SVD analyses of in-cell Pup-PA-1 and Pup-PA-3 interactions. Matrices consisting of chemical shift changes, MCSC, and intensity changes, MIC, in in-cell [*U-*^15^N] Pup peaks over the time course of PA-1 and PA-3 overexpression were analyzed^29^ to identify Pup residues involved in PA binding. The scree plots show the distribution of singular values that define the relative contribution of each binding mode to the MCSC or MIC, respectively. (a) and (d) The first binding mode contributes to 92.6% of the MCSC for Pup-PA-1 and 86.3% of the MIC for Pup-PA-3, indicating that it is a principal binding mode. A clear drop in the progression of singular values is evident after the first singular value and R^2^ values for the linear regressions are poor, 0.75 and 0.65 for (a) and (d), respectively. (b) and (c) The first binding mode contributes to 62% of the MCSC for Pup-PA-1 and 64% of the MIC for Pup-PA-3. No clear drop in singular values is evident and the R^2^ values for the linear regressions are high, 0.91 and 0.93 for (b) and (c), respectively, precluding the data from identifying principal binding modes in these cases. (e) and (f) The contribution of Pup residues to the first and second binding mode with PAs is shown in red and blue bars, respectively. There are two bars per residue. The maximum contribution of Pup residues to the second mode is used as a threshold to identify Pup residues affected by PA binding.

Both the chemical shifts and peak intensities of Pup residues were affected by ligand binding (Fig. 2 and Supplementary Fig. 1). Conventional analyses of interacting proteins, which compare the in-cell NMR spectra of the protein prior to and following full overexpression of the interactor, ignores changes in the in-cell NMR spectra that arise over the time course of the experiment from non-interactor and concentration-independent binding processes^29^. To unambiguously resolve the principal binding mode between Pup and PAs against the dynamic cellular background, we analyzed matrices composed of in-cell NMR intensity changes, MIC, and chemical shift changes, MCSC, of [*U*-^15^N]-Pup peaks over the time course of PA overexpression by using single-value decomposition, SVD^29^. In this approach, the principal binding mode between Pup and PAs is identified by an abrupt drop in the singular values (SV) of the scree plot of either MIC or MCSC (Fig. 3 and Supplementary Fig. 2). The drop results in a poor goodness of fit of linear regression, R^2^ < 0.8. Random binding of Pup to cytosol contributes to a gradual decrease in SVs, and results in R^2^ > 0.9.

The scree plot of Pup-PA-1 MCSC shows a clear drop after the first SV indicating the presence of a single principal binding mode (Fig. 3 and Supplementary Fig. 2). The corresponding plot of Pup-PA-1 MIC exhibits no drop but a gradual decrease of SVs, suggesting that, in this case, changes in intensities are not sensitive to the Pup-PA-1 binding. Interestingly, the scree plots of both Pup-PA-3 and Pup-PA-7 MIC exhibit a drop after the first SV, whereas the corresponding plots of MCSC show only a gradual decrease in SVs. Based on the first principal binding mode, Pup residues affected by PA-1 binding are Q3, D16, E35, L40, I43, D44, D45, L47, A51 and V59 (Fig. 2a and 3e); for PA-3 binding are T6, G10, G11, G12, G13, D15, D16, S21, T22, R28, R29, E30, L32, D37, E42, E48 (Fig. 2b and 3f); and for PA-7 binding are E4, T6, G10, G11, G12, G13, D14, D16, S21, T22, A23, E30, L32, D38, and D53 (Supplementary Fig. 1 and 2c).

**Figure 4:**
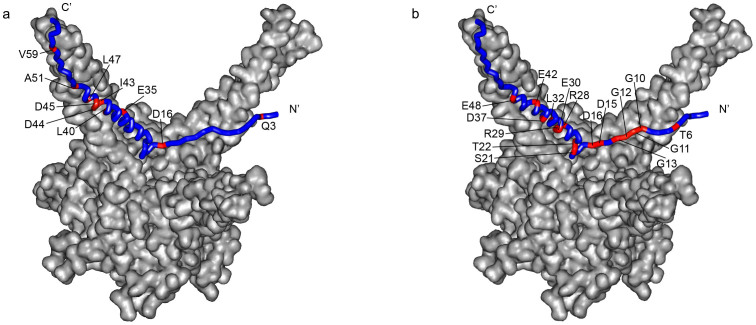
PA-1 and PA-3 bind to distinct sites on Pup. Pup residues (shown in red) identified by SVD analysis to be affected by PA-1 (a) and PA-3 (b) binding are mapped onto the Pup-Mpa complex^30^. Some labeled residues in (a) and (b) are obscured due to image orientation. The image of the Pup-Mpa structure (PDB code 3M9D) was constructed by using Modeller^45^.

Pup is an intrinsically disordered protein that forms a helical structure spanning residues 21–51 when bound to Mpa^30^. We mapped the affected residues of Pup onto the crystal structure of the Pup-Mpa complex^30^ to show how each PA interacts with Pup. For PA-1 binding, the C- terminal half of the Pup helix is most strongly perturbed (Fig. 4a); this region corresponds to the Mpa binding surface of Pup. For PA-3 binding, negatively charged residues in the N-terminal region of Pup as well as many charged residues within the Pup helix are affected (Fig. 4b). For PA-7 binding, the affected residues are similar to that of PA-3 (Supplementary Fig. 3). This result is expected due to the sequence similarity between PA-3 and PA-7 (Fig. 1b).

**Figure 5:**
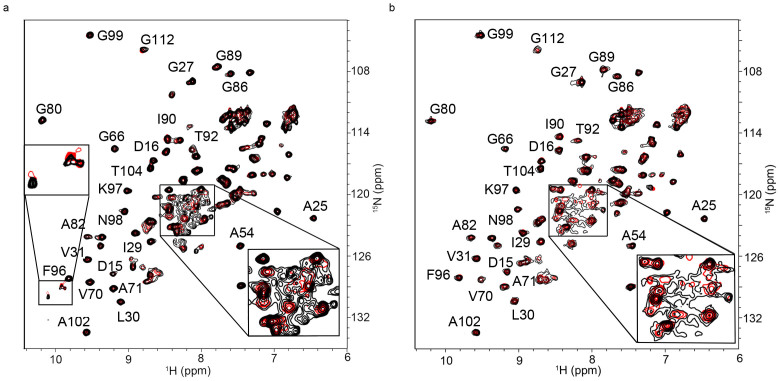
Only residues in the PA loop bind Pup. ^1^H{^15^N}-HSQC spectra of purified [*U*-^15^N] PA (red), overlaid with spectra (black) obtained after titrating with purified Pup, (a) PA-1 and (b) PA-3. Each insert shows the residues from the PA loop. Well-resolved peaks of the thioredoxin scaffold are labeled. Most peaks do not change their position or intensities, reflecting the fact that thioredoxin is a neutral PA scaffold. Only a subset of PA residues, from the PA loop (right insets), exhibit substantial broadening and chemical shift changes, indicating Pup-PA loop interactions. The chemical shifts of indole protons of tryptophans located both near and inside the PA loop of PA-1 (Figure 1) change due to the interaction with Pup (left inset). Free and Pup bound PA-3 indole protons are completely broadened and, thus, unavailable for analysis. Due to^15^N editing, only backbone amide protons and nitrogens of PA are present in the spectra.

To identify the residues of each PA that are involved in binding to Pup, we performed NMR titration experiments using bacterially expressed and purified [*U*-^15^N] PAs and bacterially expressed and purified Pup. 100 μM [*U*-^15^N] PA was titrated with 100 μM unlabeled Pup in 2 steps to yield [*U*-^15^N] PA to Pup molar ratios of 1:1 and 1:2, respectively (Fig. 5 and Supplementary Fig. 4). The thioredoxin scaffold of the PA is largely unaffected by Pup binding, supporting the idea that thioredoxin provides a neutral protein platform for aptamer loop presentation 15. NMR peaks from the aptamer loop of PAs are broadened in both free and Pup bound PAs, and thus are difficult to unambiguously assign (Figure 5). Nevertheless, we detected new peaks and changes in chemical shifts in the area of the ^1^H{^15^N}-HSQC spectrum corresponding to the aptamer loop residues suggesting that the residues located in the aptamer loop of PAs interact with Pup.

### PA-1 rescues mycobacterial proteasome substrate and inhibits *M. bovis* BCG growth

**Figure 6:**
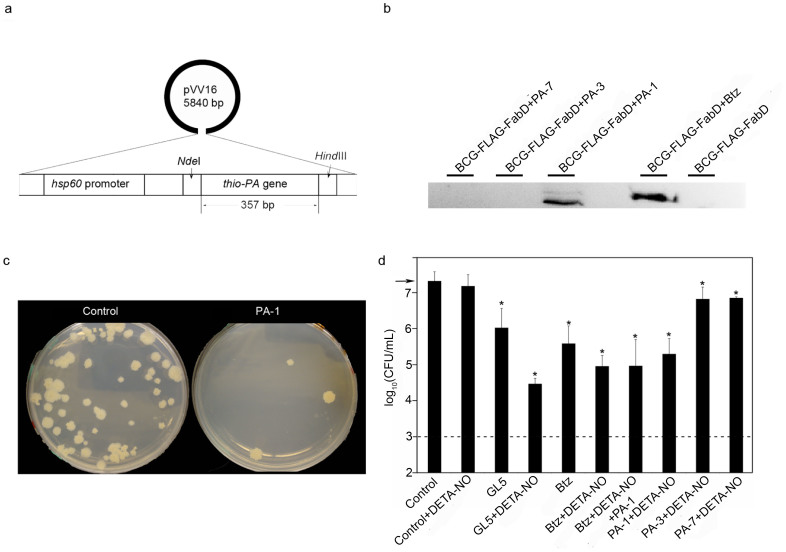
PA-1, but not PA-3 or PA-7, rescues proteasome substrate FabD from degradation and inhibits the growth of BCG. (a) Diagram of the expression shuttle vector, pVV16, used in the BCG assay. The plasmid contains an *E. coli* origin derived from pUC19, a mycobacterial origin derived from pAL5000^46^, and a constitutively active BCG *hsp60* promoter. (b) BCG-FLAG-FabD was transformed with pVV16-PA-1, -PA-3, and -PA-7 to examine the steady-state level of FLAG-tagged FabD in untreated cells. As a control, BCG was incubated with and without 50 μM Btz. Anti-groEL2 was used as a loading control. (c) BCG plates after 3 weeks of growth show a 100-fold reduction in colonies due to expression of PA-1. (d) Inhibition of BCG growth under conditions that reduce proteasome function. 50 μM Btz, GL5, and DETA- NO were used as indicated. Error bars represent standard deviations and asterisks indicate p- values <0.05 (Prism 5 Graphpad Inc.). The arrow indicates the strength of the initial inoculum. The dashed line is the lower limit of detection. All experiments were performed in triplicate.

The Bacille-Calmette-Guèrin, BCG, vaccine strain of *Mycobacterium bovis* (*M. bovis*), was used in a cell-based assay to analyze whether or not the PAs inhibit proteasome function. Each PA was cloned into a constitutively active shuttle vector, pVV16, which confers kanamycin and hygromycin resistance (Fig. 6a). FabD is a physiological substrate of the mycobacterial proteasome^31^; both endogenous *fabD* and a plasmid based FLAG epitope-tagged version of *fabD*, FLAG-*fabD*, are expressed in mycobacteria at very low levels but can be readily detected when the mycobacterial proteasome is inactive^31^. BCG expressing FLAG-*fabD* from a chromosomally integrated vector was transformed with pVV16-PA and grown in mycobacterial specific medium^32^. As a positive control, BCG with integrated FLAG-*fabD, was* grown in the presence of the proteasome inhibitor Bortezomib (Btz)^28^. Western blotting with anti-FLAG antibody showed that PA-1, but not PA-3 or PA-7, rescues FabD from proteasome degradation (Fig. 6b).

BCG was also used to analyze the effect of PAs on cell survival under nitro-oxidative stress^33^. To examine whether the PAs inhibit cell growth, BCG transformed with either null- pVV16 or pVV16-PA were grown in mycobacterial specific media^32^. We mimicked nitro-oxidative stress, which limits the replication of BCG and Mtb, by adding a nitric oxide donor, DETA-NO, to the cultures. We also grew BCG in the presence of Btz and 5-(5-methyl-2- (methylthio)thiophen-3-yl)-1,3,4-oxathiazol-2-one, GL5, another potent mycobacterial proteasome inhibitor^27^,^28^, to compare the effects of growth inhibition to each PA.

BCG cultures were grown with shaking for four days, and aliquots were plated to determine the live bacteria count. Cultures were grown for an additional three days to accumulate sufficient amounts of protein for Western blot analyses. Expression of each PA was verified by Western blotting by using an anti-thioredoxin monoclonal antibody. Native thioredoxin was not detected under control conditions (Supplementary Fig. 5).

The biological log reduction of the relative number of live microbes is expressed in terms of colony forming units, CFU/mL. After incubating plates for 3 weeks, we observed a dramatic, 100-fold or a 2 log_10_, reduction in CFU/mL for BCG expressing PA-1 grown under DETA-NO stress (Fig. 6c and 6d). Nitro-oxidative stress alone, provided by DETA-NO, had no effect on the growth of BCG (Fig. 6d). Cell growth was inhibited by the presence of proteasome inhibitors GL5 (1.0 log) and Btz (1.0–1.5 log), and, importantly, BCG viability was further reduced by the combination of GL5 and DETA-NO (2.5–3.0 log) or Btz and DETA-NO (2.0–2.5 log). These results corroborate previous studies that proteasome inhibitors limit Mtb growth under nitro- oxidative stress^27^.

Importantly, cell viability in the presence of PA-1 and DETA-NO was comparable to that of Btz and DETA-NO, suggesting that both Btz and PA-1 lead to enhancement of BCG sensitivity to nitro-oxidative stress. In contrast, PA-3 and PA-7, which interact with a segment of Pup that does not bind Mpa, in combination with DETA-NO, had only minimal effects on growth (0.5 log reduction) (Fig. 6d). Overexpression of PA-1 in the presence of Btz and DETA-NO led to no further growth inhibition beyond that of Btz and DETA-NO alone, which is consistent with PA-1 and Btz affecting the same pathway. These assays demonstrate that despite the uniformly high binding affinity of the selected PAs (Fig. 1b), the specificity of the interaction with Pup leads to distinct physiological outcomes.

## Discussion

Using CLIPs technology we isolated three peptide aptamers that bind to Pup with nanomolar affinity, PA-1, PA-3 and PA-7. The binding sites of the selected PAs are close to and even overlap each other. This fact highlights a critical advantage of Y2H selection^13^,^14^. PAs selected to bind to a given target do so without competition between the molecules in the library. These PAs would not be selected by using *in vitro* selection schemes, such as phage, yeast, or ribosomal display, where all molecules in the library compete for the same target and, consequently, only the highest affinity aptamers remain after multiple cycles of selection^34^.

Both Pup and PAs have to be over-expressed well above their physiological concentrations to be visible by in-cell NMR. Under these conditions, the cellular binding partners of Pup do not play a significant role in complex formation and can be largely ignored^35^. We also expect that Pup folding in the crowded cytosol of *E. coli* will be similar to that which occurs in mycobacteria. Most importantly, the functional assays allowed us to test the effects of PA binding to the IDP in a native physiological milieu.

In spite of similar binding affinities, the peptide aptamers identified in this screen have vastly different functional effects on rescuing a known proteasome substrate^31^, FabD, from degradation and on the survival of *M. bovis* BCG under nitro-oxidative stress. Only PA-1, but not PA-3 or PA-7, inhibits proteasome degradation of FabD in BCG (Fig. 6b). Expression of PA-1 leads to a 100-fold inhibition of growth, but PA-3 and PA-7 lead to less than a 5-fold decrease in the survival rate of DETA-NO treated cells (Fig. 6d). These effects are due to the different ways in which the PAs engage Pup, which is known to form a helical structure when bound to its target, Mpa^30^. Only the PA-1 interaction surface strongly overlaps with the Mpa helical binding surface on Pup, whereas binding of the positively charged PA-3 and PA-7 aptamers affects two highly charged segments of Pup: the N-terminal region, which is not involved in Mpa binding, and a segment of the Mpa helical binding surface on Pup. (Fig. 4 and Supplementary Fig. 3) We hypothesize that both PA-3 and PA-7 interact with Pup electrostatically with high affinity but low specificity, and can not effectively block the Pup helix from binding to Mpa.

Both of the proteasome inhibitors used in this study, Btz and GL-5, were shown to substantially increase Mtb sensitivity to nitro-oxidative stress^27^. The growth reduction caused by PA-1 with DETA-NO was comparable to that observed using Btz and DETA-NO and only 0.5 log lower than cells grown in the presence of GL5 and DETA-NO. This result suggests that PAs can be as potent as small molecules at inhibiting Mtb growth.

Drugs are widely used to inhibit enzymatic activities^27^ and it is very important to know whether these drugs have off-target effects^36^. Here we showed that known inhibitors of the Mtb proteasome in combination with PA-1 do not synergistically inhibit growth, suggesting that they act along the same biological pathway. At the same time, both GL5 and Btz are more effective than PA-1 alone in reducing the survival rate. One possible explanation is different stability of small molecular drugs compared to PA-1. Alternatively, this suggests that besides the proteasome, GL5 and Btz inhibit other enzymes important for Mtb survival.

The technology demonstrated in this work permits both screening and structural characterization of protein interactions in the crowded environment of the cytosol. This is important for IDPs, for which local structures may be partially stabilized due to macromolecular crowding^21^,^37^. Thus, selected PAs are already directed against physiologically relevant conformations, which may not be significantly populated during *in vitro* selection.

Functionally, IDPs and unstructured segments within proteins can facilitate protein-protein interactions, serve as flexible linkers between folded domains and provide convenient sites for post-translational modifications^3^. Site- and conformation-specific binding of PAs to IDPs or unstructured segments uniquely affect these functions, either by blocking access to the protein interaction sites and the sites of post-translational modification, or by decreasing the flexibility of critical sites in the linkers. The availability of PAs selected to bind to specific sites on IDPs allows unprecedented opportunity to analyze the functional consequences of IDP interactions with atomic precision in live cells.

## Methods

### Reagents and chemicals

Restriction enzymes, *Taq* DNA Polymerase, and Phusion DNA Polymerase were from New England BioLabs. *Pfu* DNA Polymerase was from Agilent Technologies. All other chemicals used were reagent grade or better.

### Yeast strains

Matchmaker Gold yeast-hybrid system 2 and vectors, pGBKT7 and pGADT7, were obtained from Clontech. Yeast strain Y2HGold (MATa, trp1-901, leu2-3, 112, ura3-52, his3-200, gal4Δ, gal80Δ, LYS2:: GAL1_UAS_–Gal1_TATA_–His3, GAL2_UAS_–Gal2_TATA_–Ade2, URA3:: MEL1_UAS_– Mel1_TATA_, AUR1-C MEL1) and Y187 (MATα, ura3-52, his3-200, ade2-101, trp1-901, leu2-3, 112, gal4Δ, gal80Δ, met-, URA3:: Gal1_UAS_–Gal1_TATA_–LacZ, MEL1) were used for peptide screening and grown in yeast peptone dextrose adenine (YPDA) growth medium. Four reporter genes, *AUR1-C*, *ADE2*, *HIS3*, and *MEL1*, under the control of GAL4 upstream activating sequences (UASs) and TATA boxes, were used to select PAs and eliminate false positives. *AUR1-C* confers strong resistance to the highly toxic antibiotic, Aureobasidin A (Aur A). *ADE2* and *HIS3* provide metabolic selection, and *MEL1*, which encodes α-galactosidase, is used for X- α-galactosidase (X-α-gal) selection.

### Plasmid Construction

The peptide aptamer (PA) library generated in our lab was used to screen against Pup^13^. The library contains approximately 3*10^10^ peptide aptamers cloned into pGADT7 to yield a pGADT7-thioredoxin-peptide aptamer library construct in which an 8-amino-acid randomized peptide is inserted into a constrained loop of D26A, K57Q thioredoxin^13^. The thioredoxin is fused in frame with amino acids 768–881 of the GAL4 activating domain (AD). The plasmid has a hemagglutinin (HA) epitope tag, a 2 μ origin, and confers ampicillin resistance for selection in *E. coli* and LEU2 for selection in yeast.

DNA encoding Pup (64 aa) was PCR amplified from *Mtb* genomic DNA using *Taq* DNA Polymerase and oligonucleotides 5′-TTTTTTCATATGGCGCAAGAGCAGACCAAGC-3′ and 5′- TTTTTTGTCGACTCACTGTCCGCCCTTTTGG-3′, which contain flanking 5′-*Nde*I and 3′-*Sal*I restriction sites. The restriction-digested PCR product was ligated into expression vector pGBKT7 to yield pGBKT7-Pup in which *Pup* is fused in frame with amino acids 1–147 of the GAL4 DNA binding domain (DNA-BD). The expressed protein contains an N-terminal c-Myc epitope tag and confers kanamycin resistance for selection in *E. coli* and TRP3 for selection in yeast.

DNA encoding Mpa (613 aa) was PCR amplified from *Mycobacterium smegmatis* genomic DNA using Phusion DNA Polymerase and oligonucleotides 5′- AAAAAAGGTACCATGAGTGAGTCAGA-3′ and 5′-AAAAAGGTACCCAGGTACTGGCCCAG-3′, which contains flanking 5′- and 3′-*Kpn*I restriction sites. The restriction-digested PCR product was ligated into pGADT7 to yield pGADT7-Mpa in which *Mpa* is fused to a GAL4 activating domain. The expressed protein contains a C-terminal hemagglutinin (HA) epitope tag and ampicillin resistance for selection in *E. coli* and LEU2 for selection in yeast.

DNA encoding each individual peptide aptamer was PCR amplified from pGADT7 using *Taq* Polymerase and oligonucleotides 5′-CGGGGCCATCCTCGTCGCTTTCTGG-3′ and 5′- GGTTTTGATCGATGTTCAGTTGTGCA-3′. *Pfu* DNA Polymerase and PCR products were used to clone peptide aptamers into a modified pBAD expression vector, which confers ampicillin resistance. The resulting plasmids, pBAD-PA, express N-terminal 6xHis-tagged scaffold protein, thioredoxin.

DNA encoding each peptide aptamer was PCR amplified using Phusion DNA Polymerase and oligonucleotides 5′-TTTTTTCATATGGGCCATAAAATTATT-3′ and 5′- CCCAAGCTTACGCCAGGTTAGCGTCG-3′, which contain flanking 5′-*Nde*I and 3′-*Hind*III restriction sites. The restriction-digested PCR product was ligated into pVV16 (BEI Resources) to yield pVV16-PA. The pVV16 affords heterologous protein expression under constitutively active hsp60 promoter and confers kanamycin and hygromycin resistance for selection in *E. coli* and mycobacterial strains. Each aptamer was handled in the same manner.

To make the FLAG-tagged mycobacterial proteasome substrate, *fabD* was amplified by PCR from BCG genomic DNA using primers encoding the FLAG epitope (MDYKDDDDKI), 5′-GGGTGCGAGCAACGCAATCATCTTATCGTCGTCATCCTTGTAATCCATGGTACC-3′ and 5′-GGATCCTTATAGGTTTGCCAGCTCGTCCAGGTC-3′, which contain flanking 5′- *KpnI* and 3′-*BamHI* restriction sites. The restriction-digested PCR product was ligated into the BCG plasmid pMBC1260. pMBC1260 is a single copy plasmid that integrates into the attB site on the BCG chromosome and affords heterologous protein expression off of the constitutively active *Rv0805* promoter and confers kanamycin resistance for selection in *E. coli* and mycobacterial strains.

### Verification of reporter gene activation

Plasmid pGBKT7-Pup (bait) was transformed into yeast strain Y2HGold and plasmid pGADT7-Mpa (prey) was transformed into yeast strain Y187. Transformants were mated on YPDA agar plates and mated transformants were selected on synthetic dropout media (SDM) lacking tryptophan and leucine, SDM lacking tryptophan, leucine, and histidine, SDM lacking tryptophan, leucine, and adenine, SDM lacking tryptophan and leucine supplemented with X-α- gal, and SDM lacking tryptophan and leucine supplemented with Aur A, to verify the activation of reporter genes by these constructs.

### Library transformation and screening

Plasmid pGBKT7-Pup was used as bait to screen CLIPs^13^ by using the Matchmaker Gold yeast-hybrid system 2 protocol. The CLIPs was transformed into yeast strain Y2HGold harboring pGBKT7-Pup by using the lithium acetate method. Transformants were selected initially for growth on ade^−^ trp^−^ leu^−^ plates. Positive clones were streaked onto trp^−^ leu^−^ his^−^ plates; ade^−^ trp^−^ leu^−^ plates; trp^−^ leu^−^ plates supplemented with X-α-gal; and trp^−^ leu^−^ plates supplemented with Aur A, to test the stringency of the interaction with Pup.

### Expression and purification of aptamers for *in vitro* assays

For fluorescence titration experiments, *E. coli* strain DH10B was transformed with pBAD- PA and grown in M-505^38^ auto-inducing medium containing 0.25% L-arabinose, and 150 mg/L of carbenicillin. All PA plasmids were treated in the same manner. For [*U*-^15^N] labeling of PAs, *E. coli* strain Origami B (Novagen) was transformed with pBAD-PA and grown in N-505^38^ auto- inducing medium containing 2.66 g/L of [*U*-^15^N] ammonium chloride as the sole nitrogen source, 0.25% L-arabinose, and 150 mg/L of carbenicillin. Cultures were grown at 37°C for 20–26 h. Cells were harvested, re-suspended in extraction buffer (50 mM sodium phosphate, pH 7.0, 300 mM sodium chloride, 6 M guanidine hydrochloride) and sonicated. The lysate was cleared by centrifugation, and the supernatant was loaded onto a pre-equilibrated TALON column (Clontech). The column was washed with 10 column volumes (CV) of extraction buffer, 10 CV of extraction buffer containing 0.4% octyl phenol ethoxylate (Triton X-100, Baker), and again with 10 CV of extraction buffer. Protein was eluted with 10 CV of 1× imidazole elution buffer (45 mM sodium phosphate, pH 7.0, 270 mM sodium chloride, 5.4 M guanidine hydrochloride, 150 mM imidazole). Eluted protein was concentrated to 2–2.5 mL by using an Amicon Ultra-15 centrifugal filter (Millipore) with a 3 kDa molecular weight cutoff (MWCO), and supplemented with 50 μL of 0.5 M EDTA, 6 mL of TSP buffer (20 mM sodium phosphate, pH 7.5, 100 mM sodium thiosulfate) and 1 mL of glycerol. Protein samples were dialyzed against 1 L of 50 mM sodium phosphate, pH 8.0, 100 mM sodium chloride, 0.5 M guanidine hydrochloride, 2 mM EDTA, and 10% glycerol, for 5–10 hours at 4°C in a 1 kDa MWCO dialysis bag (Spectrum Laboratories, Inc.). Samples were further dialyzed against 2 L of TSP buffer supplemented with 10% glycerol, for 10 hours at 4°C. Finally, peptide aptamers were concentrated by using an Amicon Ultra-15 centrifugal filter with a 3 kDa MWCO, exchanged into TSP buffer and stored on ice. The peptide aptamers were estimated to be >95% pure by Coomassie-stained SDS- PAGE.

### Expression and purification of Pup for *in vitro* assays

*E. coli* strain BL21(DE3) Codon+ (Novagen) was transformed with pTM-Pup, which encodes a tryptophan leader sequence (trpL) before Pup^39^. Cells were grown to an OD_595_ of 0.5 in minimal medium (M9) containing 35 mg/L of kanamycin, induced with 0.5 mM isopropyl 1- thio-β-d-galactopyranoside (IPTG), and grown overnight at 37°C. Cells were harvested, re- suspended in 20 mM HEPES-Na, pH 7.0 buffer containing 8 M urea, and sonicated. The lysate was cleared by centrifugation, and the supernatant was loaded onto a nickel-nitrilotriacetic acid- agarose (Ni-NTA) column (Qiagen). The column was washed with 10 CV of wash buffer (20 mM HEPES-Na buffer, pH 7.0, containing 8 M urea), and the protein was eluted with elution buffer (20 mM phosphate buffer, pH 4.0 containing 8 M urea). Eluted protein was concentrated to 2–2.5 mL by using an Amicon Ultra-15 centrifugal filter (Millipore), with a 3 kDa MWCO. The concentrated sample was dialyzed against 1 L of 10 mM sodium phosphate buffer, pH 6.5, for 3–4 h at 4°C in a 1 kDa MWCO dialysis bag (Spectrum Laboratories, Inc.). Formic acid was added to the resultant sample to a final concentration of 70% and the N-terminal 6xHis-tag and trpL^39^ were removed by cyanogen bromide cleavage at room temperature for 1 h. The sample was cleared by centrifugation and placed in a 1 kDa MWCO dialysis bag (Spectrum Laboratories, Inc.) for dialysis against 4 L of 10 mM potassium phosphate buffer, pH 7.0, at room temperature. The dialysis buffer was changed every 3–4 hours or until a precipitate formed. The sample was cleared by centrifugation, concentrated by using an Amicon Ultra-15 centrifugal filter with a 3 kDa MWCO and loaded onto an anion exchange column (Amersham Biosciences). The protein was eluted with 30 mL of 10 mM potassium phosphate buffer, pH 7.0, using a 0–1 M NaCl gradient. The fractions containing eluted protein were concentrated by using an Amicon Ultra-4 centrifugal filter with a 3 kDa MWCO and the final protein was stored on ice. Protein was estimated to be >95% pure by Coomassie-stained SDS-PAGE.

### Fluorescence titrations

Native tryptophan fluorescence experiments were conducted using a Horiba Jobin Yvon Fluorolog spectrofluorometer equipped with a Perkin Elmer 4 × 4 mm quartz cuvette. 100 nM aptamer solutions were individually titrated from 0.01–2 μM with Pup in 500 μL of TSP buffer. The excitation and emission wavelengths were 280 nm and 352 nm, respectively. Dissociation constants, K_d_, were estimated from the changes in peak fluorescence intensities as a function of Pup concentration by using Prism 5 software (GraphPad). Data were fit to the equation, (F- F_0_)/F_max_ = [Pup]/(K_d_+[Pup]) where F is the fluorescence intensity at a given Pup concentration, F_0_ is the fluorescence intensity of the blank, and F_max_ is the maximum fluorescence intensity.

### Sequential over-expression of ^15^N-Pup and aptamers for STINT-NMR

Plasmids pRSF-Pup and pBAD-PA were co-transformed into *E. coli* strain BL21(DE3) Codon+ (Novagen) for sequential over-expression. To evaluate the interaction between Pup and the peptide aptamers, [*U*-^15^N] Pup was overexpressed for two hours followed by 2, 4, 6, and 16 hours over-expression of unlabeled PA. Each PA was handled in the same manner.

For [*U*-^15^N] labeling, cells were grown in minimal medium (M9) containing 100 mg/L of ampicillin, 35 mg/L of kanamycin, and 1 g/L of [*U*-^15^N] ammonium chloride as the sole nitrogen source. Pup expression was induced with 0.5 mM IPTG at an OD_595_ of 0.5 and grown for 2 h at 37°C. A 100 mL sample of culture was collected, centrifuged, washed twice with 50 mL of 10 mM potassium phosphate buffer, pH 6.5, re-suspended in 500 μL of NMR buffer and 10% D_2_O, and used immediately for NMR analysis. A control sample of [*U*-^15^N] Pup was collected to assess the extent of over-expression. The remainder of the culture was centrifuged, washed twice with M9 salts, re-suspended in fresh unlabeled minimal medium containing 100 mg/L of ampicillin and 35 mg/L of kanamycin. PA expression was induced with 0.25% L-arabinose (w/v) and grown overnight at 37°C. A 100 mL sample of culture was collected, centrifuged, washed twice with 50 mL of 10 mM potassium phosphate buffer, pH 6.5, re-suspended in 500 μL of NMR buffer and 10% D_2_O, and used immediately for subsequent NMR analysis.

### NMR Spectroscopy

NMR experiments were performed on Bruker Avance III spectrometers equipped with a cryoprobe, operating at ^1^H frequencies of 500 MHz and 700 MHz. All spectra were collected at 298 K, which yielded high quality spectra of Pup. We used a Watergate version of the ^1^H{^15^N}- edited heteronuclear single quantum coherence (HSQC) experiment recorded with 64 transients as 512x64 complex points in proton and nitrogen dimensions, respectively, apodized with a squared cosine-bell window function and zero-filled to 1024 × 128 points prior to Fourier transformation. The corresponding sweep widths were 12 and 35 ppm in the ^1^H and ^15^N dimensions, respectively. The spectra were processed by using the program TOPSPIN (Bruker, Inc) and the program CARA^40^,^41^ was used for spectral analysis. Chemical shifts of in-cell [*U*-^15^N] Pup were assigned^42^. Thioredoxin scaffold chemical shifts were obtained from Reverdatto *et al*^13^. To reassign the [*U*-^15^N] Pup peaks that changed position due to complex formation we assumed minimum chemical shift changes^43^, calculated as ΔΩ = ((ΔΩ_HN_)^2^ + (0.25 * ΔΩ_N_)^2^)^1/2^, where Ω_NH_ and Ω_N_ represent amide hydrogen and nitrogen chemical shifts, respectively. Peak intensity changes were calculated as: (I/I_ref_)_free_ - (I/I_ref_)_complex_, where *I* is an individual peak intensity and I_ref_ is the peak intensity of a glutamine at 7.45 ppm and 112.5 ppm in the proton and nitrogen dimensions, respectively, that does not shift during titration.

The same peak served as *I_ref_* in the spectra of free [*U*-^15^N] Pup and in peptide aptamer complexes. The data were represented by an m × n matrix of either chemical shift changes, MCSC, or peak intensities changes, MIC, in which the column index, n, represents different time points in the overexpression of PA, corresponding to increasing concentration, and the row index, m, represents the chemical shift or intensity changes of amino acid peaks on Pup, respectively. SVD analyses of the matrices were performed as described^29^ without modification to identify principal in-cell binding modes of PAs to Pup. The significance of a particular mode was assessed by its singular value. The maximum contribution of Pup residues to the second mode was used as a threshold to identify Pup residues affected by PA binding.

### *M. bovis* proteasome substrate inhibition and growth assay

Plasmid pMBC1260-FLAG-*fabD* was transformed into *M. bovis* BCG (Pasteur strain, Trudeau Institute). Approximately 1.5 μg of DNA was added to 50 μL of cells in a 0.2 cm electroporation cuvette and DNA was introduced using a Gene Pulser (Bio-Rad) set at 2.5 kV, 25 μF, with the pulse controller resistance set at infinity. Following electroporation, the sample was immediately diluted with 2 mL of mycomedium^32^, Middlebrook 7H9 medium (Difco), supplemented with 0.5% (v/v) glycerol, 10% (v/v) oleic acid-albumin-dextrose-catalase (OADC) and 0.05% (v/v) Tween 80), and incubated at 37°C overnight. Cells were plated on Middlebrook 7H10 (Difco) plates supplemented with 10% OADC, 0.01% (w/v) cycloheximide, and 25 μg/mL of kanamycin and incubated for 3 weeks at 37°C. Colonies were grown in 5 mL of mycomedium for 5–7 days at 37°C for PCR screening and seed stocks. Each pVV16-PA was introduced into BCG under 25 μg/mL of kanamycin selection in the same manner. Each pVV16- PA was introduced into BCG with integrated FLAG-*fabD* under 100 μg/mL of hygromycin selection in the same manner.

To monitor the growth of BCG during the expression of each PA, cultures containing 2.5*10^6^ CFU/mL were grown for 4 days under various conditions at 37°C, in the presence of 25 μg/mL of kanamycin. Vector only BCG was used as a negative control. 50 μM bortezomib (Btz) (Sigma-Aldrich) and 50 μM 5-(5-methyl-2-(methylthio)thiophen-3-yl)-1,3,4-oxathiazol-2-one (GL5) were used as positive controls for inhibition. BCG was assayed in the presence of 50 μM 2,2-(hydroxynitrosohydrazino)-bis-ethanamine (DETA-NO), 50 μM GL5 with and without 50 μM DETA-NO, 50 μM Btz with and without 50 μM DETA-NO, and 50 ∝M Btz, 50 ∝M DETA- NO and PAs. Each culture was replenished with GL5, Btz, and DETA-NO after two days of growth^44^. Triplicates of each culture were grown and serial dilutions ranging from 10^−2^ to 10^−5^ were plated in triplicate and incubated for 3 weeks at 37°C to enumerate the surviving CFU. Mean ±S.D. values are reported. Statistical comparisons between untreated BCG and treated BCG were performed by using Student's t test.

### Western blotting.

BCG cultures were grown for seven days to late log phase for Western blot analyses. To inhibit the BCG proteasome, cultures were grown for 5 days without and 2 days with 50 μM Btz. Cells were collected, washed with phosphate buffered saline (PBS) buffer containing protease inhibitor, re-suspended at 0.25 mg/μL in Tris-sodium dodecyl sulfate (Tris/SDS) buffer, 0.3% (w/v) SDS, 50 mM Tris-HCl [pH 8.0], and sonicated. Sonicated sample were frozen and thawed 10 times and the sonication was repeated to lyse the bacteria. Lysates were heated for 5 min at 37°C after adding 1% SDS and 0.0125% β-mercaptoethanol. Lysates were cleared by centrifugation and stored at −80°C for subsequent analysis. Lysates were immunoblotted and probed for FLAG-FabD or thioredoxin with an anti-FLAG horseradish peroxidase (HRP)- conjugated polyclonal antibody (Cell Signaling) or anti-thioredoxin monoclonal antibody (Novagen), respectively. For anti-thioredoxin, HRP-conjugated goat anti-mouse IgG (Amersham Pharmacia Biotechnology) was used to identify sites of binding by the primary antibody. Blots were incubated in SuperSignal West Pico Chemiluminescent Substrate (Thermo Scientific) for 15 min and imaged on a ChemiDoc XRS+ System (Bio-Rad) by using Quantity One software. Membranes were stripped of bound antibodies and re-probed with anti-groEL2 (Abcam) for loading controls.

## Author Contributions

A.S. and K.M. designed the experiments. J.C., D.B. and A.S. wrote the main manuscript text. J.C., C.D., S.M., S.R. and E.S. performed the experiments and prepared the figures. All authors reviewed the manuscript.

## Supplementary Material

Supplementary Information Supplementary Information
