# Supplementary material for: Caught in Action: Selecting Peptide Aptamers Against Intrinsically Disordered Proteins in Live Cells
Source: Sci Rep. 2015 Mar 24;5:9402. doi: 10.1038/srep09402 (PMC4371151; doi:10.1038/srep09402)
Supplement: Supplementary Information [file srep09402-s1.pdf]

Supplementary Information

# Caught in Action: Selecting Peptide Aptamers Against Intrinsically Disordered Proteins in Live Cells

Jacqueline D. Cobbert[1], Christopher DeMott[1], Subhabrata Majumder[1], Eric A. Smith[2], Sergey Reverdatto[1], David S. Burz[1], Kathleen A. McDonough[2], Alexander Shekhtman[1*]

[1] Department of Chemistry, University at Albany, Albany, NY
[2] Wadsworth Center, NY State Department of Health, Albany, NY

**List of Figures.**

**Supplementary Figure 1.** In-cell NMR of [$U$-$^{15}$N] Pup-PA-7.

**Supplementary Figure 2.** SVD analysis of Pup-PA-7 binding.

**Supplementary Figure 3.** Pup residues affected by the interaction with PA-7 are similar to that of PA-3.

**Supplementary Figure 4.** Only the PA loop is involved in PA-7 binding to Pup.

**Supplementary Figure 5.** Expression of PAs in BCG.

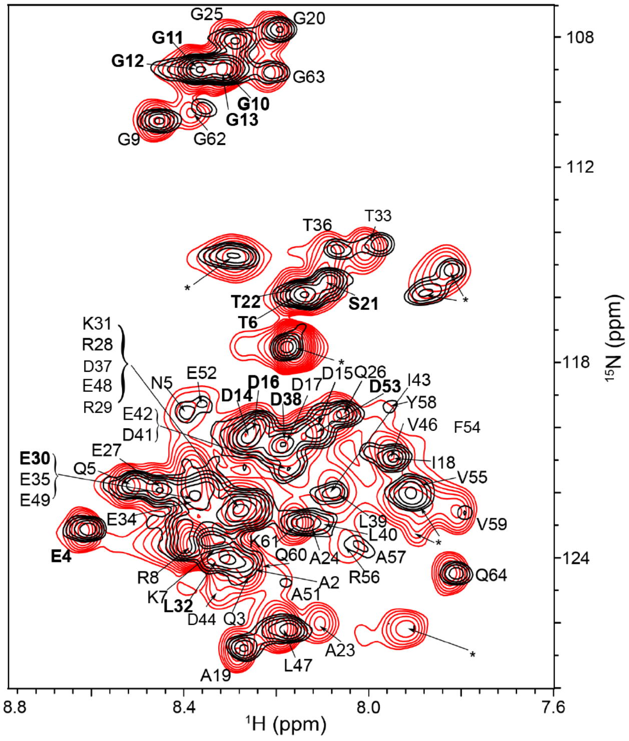


**Supplementary Figure 1. In-cell NMR of [*U*-$^{15}$N] Pup-PA-7.** (a) $^{1}$H{$^{15}$N}-HSQC spectrum of *E. coli* after 2 h of [*U*-$^{15}$N] Pup over-expression (red), overlaid with spectrum (black) of *E. coli* after 2 h of [*U*-$^{15}$N] Pup over-expression followed by approximately 16 h over-expression of PA-7. Due to $^{15}$N editing, only backbone amide protons and nitrogens of Pup are present in the spectrum. Most peaks do not change their positions indicating that only a subset of Pup residues interact with PA-7 (bold). The sharp peaks in the spectrum, which correspond to various metabolites of [*U*-$^{15}$N] ammonium ion, are labeled with asterisks.

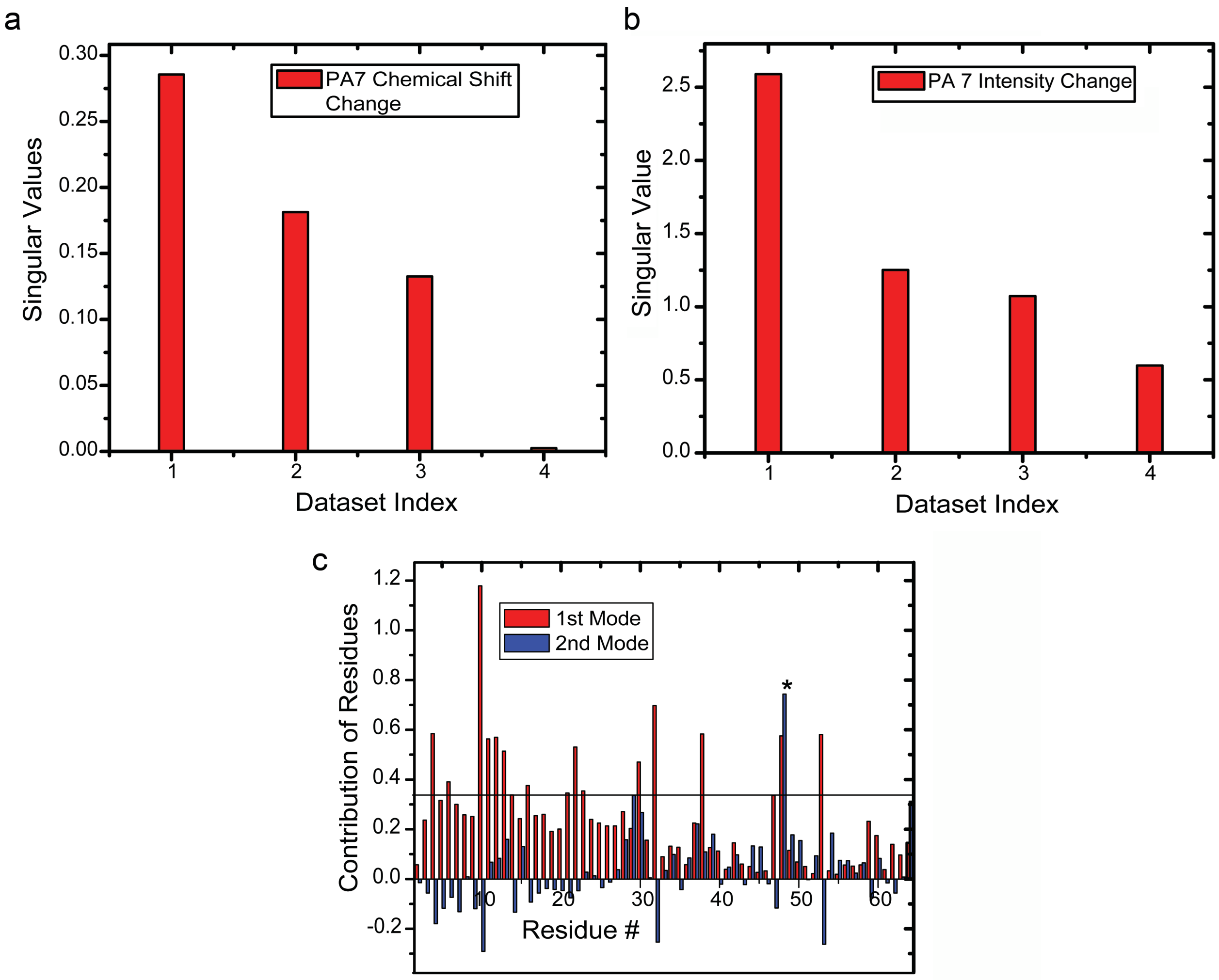


**Supplementary Figure 2. SVD analysis of Pup-PA-7 binding.** Matrices consisting of either chemical shift changes, MCSC, or intensities changes, MIC, in the in-cell [*U*-$^{15}$N] Pup peaks over the time course of PA-7 overexpression were analyzed[1] to identify Pup residues involved in PA binding. The scree plots (**a** and **b**) show the distribution of singular values that define the relative contribution of each binding mode to the MCSC or MIC, respectively. **(a)** The first binding mode of Pup-PA-7 contributes to 61.8% of MCSC, respectively, with no clear drop in singular values, precluding us from identifying principal binding modes in this case. The $R^2$

value of the scree plot linear regression is 0.97. **(b)** The first binding mode of Pup-PA-7 contributes to 68.5% of MIC, respectively, indicating that it is a potential principal binding mode. A clear drop in the progression of singular values is evident after the first singular value. The $R^2$ value of the scree plot linear regression is 0.79. **(c)** The contribution of Pup residues to the first and second binding modes with PA-7 is shown in red and blue bars, respectively. The maximum contribution of Pup residues to the second mode is used as a threshold to identify Pup residues affected by PA-7 binding. Proximity to a metabolite peak generates an anomalously large contribution to the second binding mode by E48 indicated by an asterisk.

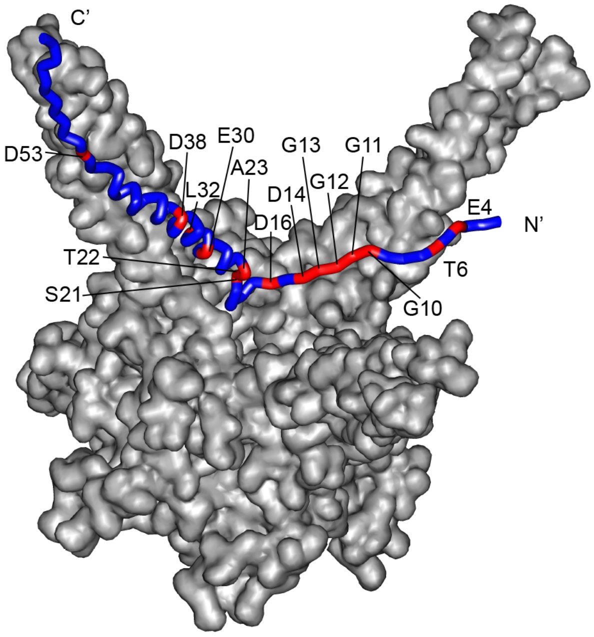


**Supplementary Figure 3**. **Pup residues (shown in red) affected by the interaction with PA-7 are similar to that of PA-3.** Based on the SVD analysis of in-cell NMR data, the negatively charged N-terminal tail and a few residues of the α-helix that interact with Mpa are affected by the binding of PA-7. Some labeled residues are obscured due to image orientation. The image of the Mpa-Pup structure (PDB code 3M9D[2]) was constructed by using Modeller[3].

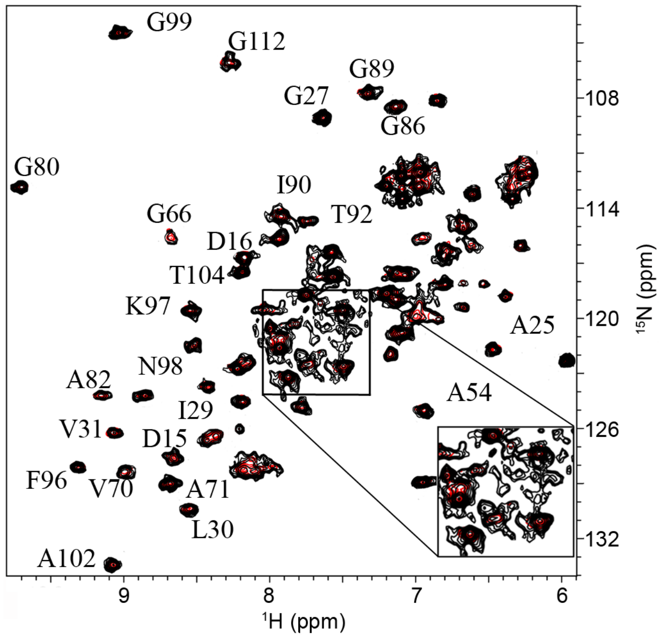


**Supplementary Figure 4. Only the PA loop is involved in PA-7 binding to Pup.** A $^{1}H\{^{15}N\}$-HSQC spectrum of purified [*U*-$^{15}$N]-PA (red), overlaid with a spectrum (black) obtained after titrating with purified Pup. The insert shows the residues from the PA-7 loop. Well-resolved peaks of the thioredoxin scaffold are labeled. Most peaks do not change their positions or intensities reflecting the fact that thioredoxin is a neutral PA scaffold. Only a subset of PA residues, from the PA loop, exhibit substantial or complete broadening of peaks, indicating Pup-PA-7 loop interaction. Due to $^{15}$N editing, only backbone and side chain amide protons and nitrogens of PA are present in the spectra.

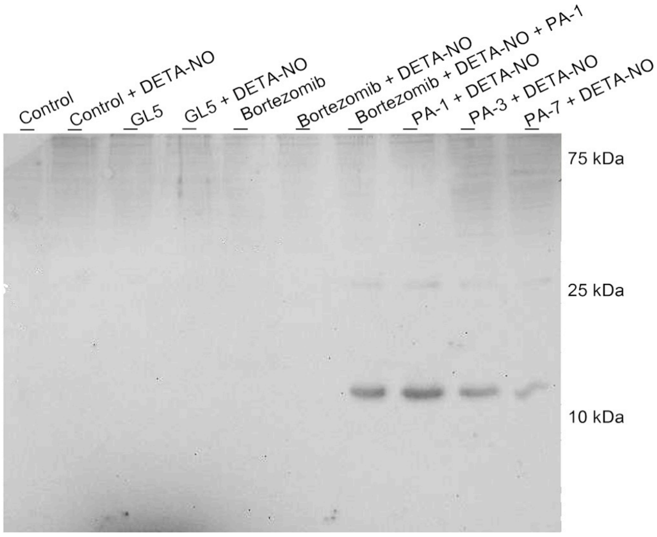


**Supplementary Figure 5. Expression of PAs in BCG.** Expression of PAs in BCG was verified by Western blot analysis. Lysates from *M. bovis* BCG cultures were probed for thioredoxin. Thioredoxin was not detected in the control cultures (lanes 1-6) and was detected at ~12 kDa in the cultures in which PAs were expressed (lanes 7-10).


1. Majumder, S., DeMott, C.M., Burz, D.S. & Shekhtman, A. Using singular value decomposition to characterize protein-protein interactions by in-cell NMR spectroscopy. *Chembiochem* **15**, 929-33 (2014).
2. Wang, T., Darwin, K.H. & Li, H. Binding-induced folding of prokaryotic ubiquitin-like protein on the Mycobacterium proteasomal ATPase targets substrates for degradation. *Nat Struct Mol Biol* **17**, 1352-7 (2010).
3. Eswar, N., Eramian, D., Webb, B., Shen, M.Y. & Sali, A. Protein structure modeling with MODELLER. *Methods Mol Biol* **426**, 145-59 (2008).
